# Serrated lesions of the colon and rectum: Emergent epidemiological data and molecular pathways

**DOI:** 10.1515/med-2020-0226

**Published:** 2020-11-09

**Authors:** Michele Sacco, Fatima Domenica Elisa De Palma, Elia Guadagno, Mariano Cesare Giglio, Roberto Peltrini, Ester Marra, Andrea Manfreda, Alfonso Amendola, Gianluca Cassese, Vincenza Paola Dinuzzi, Francesca Pegoraro, Francesca Paola Tropeano, Gaetano Luglio, Giovanni Domenico De Palma

**Affiliations:** Department of Clinical Medicine and Surgery, University of Naples Federico II via Sergio Pansini, 5 – 80131, Naples, Italy; CEINGE Biotecnologie Avanzate s.c.ar.l., Via Comunale Margherita, 80131, Naples, Italy; Department of Molecular Medicine and Medical Biotechnologies, University of Naples Federico II, via Sergio Pansini, 5 – 80131, Naples, Italy; Department of Advanced Biomedical Sciences, Pathology Section, University of Naples Federico II, Naples, Italy

**Keywords:** colorectal cancer, serrated adenomas, molecular pathways, interval cancer, BRAF

## Abstract

In 2010, serrated polyps (SP) of the colon have been included in the WHO classification of digestive tumors. Since then a large corpus of evidence focusing on these lesions are available in the literature. This review aims to analyze the present data on the epidemiological and molecular aspects of SP. Hyperplastic polyps (HPs) are the most common subtype of SP (70–90%), with a minimal or null risk of malignant transformation, contrarily to sessile serrated lesions (SSLs) and traditional serrated adenomas (TSAs), which represent 10–20% and 1% of adenomas, respectively. The malignant transformation, when occurs, is supported by a specific genetic pathway, known as the serrated-neoplasia pathway. The time needed for malignant transformation is not known, but it may occur rapidly in some lesions. Current evidence suggests that a detection rate of SP ≥15% should be expected in a population undergoing screening colonoscopy. There are no differences between primary colonoscopies and those carried out after positive occult fecal blood tests, as this screening test fails to identify SP, which rarely bleed. Genetic similarities between SP and interval cancers suggest that these cancers could arise from missed SP. Hence, the detection rate of serrated-lesions should be evaluated as a quality indicator of colonoscopy. There is a lack of high-quality longitudinal studies analyzing the long-term risk of developing colorectal cancer (CRC), as well as the cancer risk factors and molecular tissue biomarkers. Further studies are needed to define an evidence-based surveillance program after the removal of SP, which is currently suggested based on experts’ opinions.

## 1 Introduction

The prevention of colorectal cancer (CRC) is carried out through the identification and removal of premalignant lesions [1]. The last decade has witnessed the appearance of new protagonists in this scenario, the serrated polyps (SP), which are reported to be at the origin of 15–30% of all CRCs [2,3].

The term “serrated adenoma” was coined by Longacre and Fenoglio-Preiser in 1990 to describe a new type of colorectal polyp presenting with peculiar characteristics [4]. In this lesion, the colonic mucosa presented a saw-toothed appearance, similar to hyperplastic polyps (HPs), but with some cytological atypia and, in some cases, dysplasia [4–7]. In 1996, Torlakovic and Snover described the serrated sessile adenomas/polyps and traditional serrated adenoma (TSA) [6].

In 2010, the inclusion of serrated lesions in the WHO classification of digestive tumors [8] let grow up the attention of the scientific community about the malignant potential of SP, which present peculiar characteristics. In contrast to the well-known conventional adenomas, SP usually presents as flat lesions with indistinct borders and can have rapid growth. Also, SP show genetically peculiarities, being characterized by several mutations and changes in phenotype, which occur within a specific pathway [5,9–13].

In the last years, there has been widespread of literature focusing on SP. This review aimed to analyze the available data on the epidemiological and molecular aspects of these lesions.

## 2 Classification

**Figure 1:**
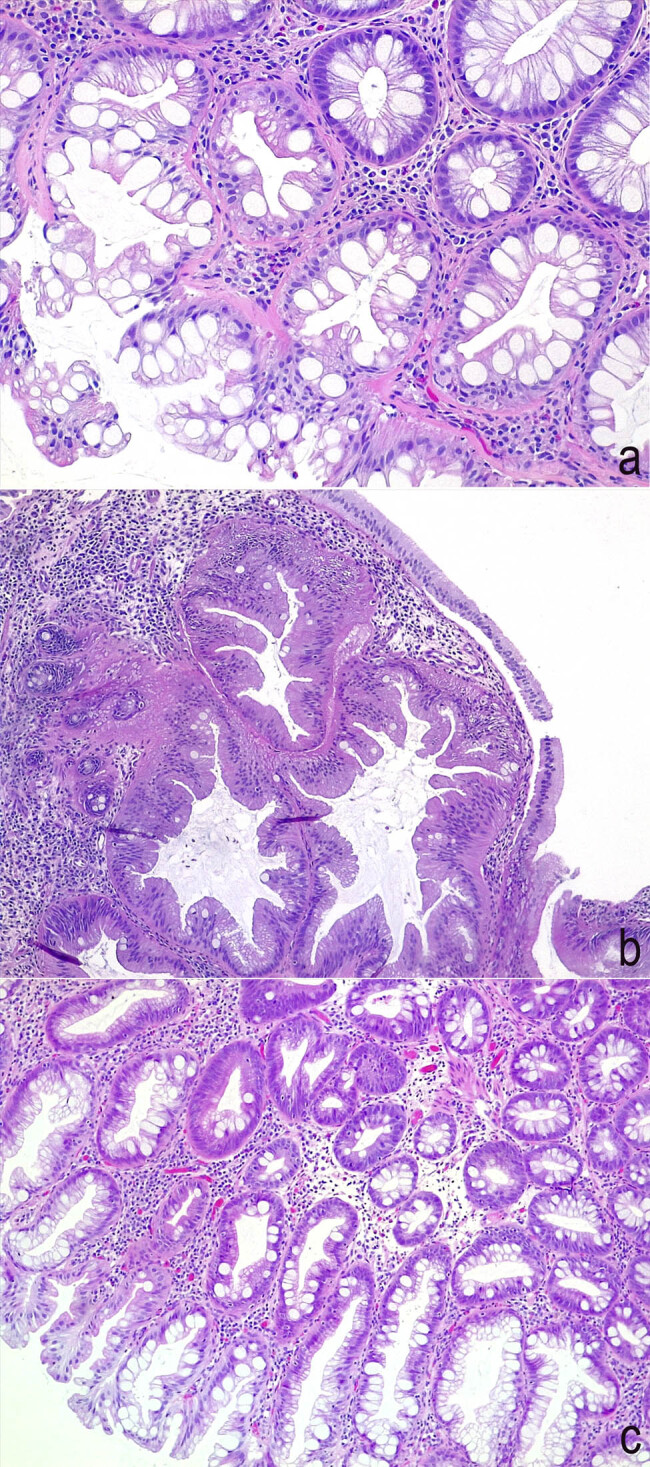
Histopathologic features of serrated lesions. (a) Goblet cell-rich hyperplastic polyp. The crypts show the typical sawtooth architecture, which is more evident superficially and contain a large number of goblet cells. (Hematoxylin and eosin stain, 200× magnification.) (b) Sessile serrated lesion. Deep serration and basal crypt dilation can be observed in the present field. (Hematoxylin and eosin stain, 100× magnification.) (c) Sessile serrated lesion with dysplasia. Besides crypt serration and dilation, low-grade dysplastic foci can be observed in the present case. (Hematoxylin and eosin stain, 100× magnification.)

The 2019 WHO Classification recognizes four subtypes of SP [8]: HPs, sessile serrated lesions (SSLs), sessile serrated lesions with dysplasia (SSLsD), and traditional serrated adenomas (TSAs) [14]. The new classification strongly discouraged the use of the term adenoma or polyp previously used to indicate nondysplastic SSLs. Some histopathologic features of SPs are shown in Figure 1.

### 2.1 Hyperplastic polyps

HP is the most common SP, accounting for 70–90% of all SP [15]. HPs are considered benign lesions, with a minimal or null risk of progression to CRC. In the course of a colonoscopy, HPs appear as smooth, symmetric, and pale lesions and are mainly found in the rectum and sigmoid colon. HPs are characterized by the presence of straight crypts, rising perpendicularly from the *muscolaris mucosae*. They have a jagged infolding crypt epithelium, more pronounced near the luminal surface, which gives them a “serrated” appearance (Figure 1a) [5,16].

According to the mucin content of the epithelial cells, HPs are subdivided into three subtypes: microvesicular HPs (MVHP), whose cells have a vacuolated cytoplasm with small mucin droplets; goblet cell HPs (GCHP), with large mucin apical vesicles, and mucin-poor HPs (MPHP), with scarce cytoplasm mucin [9,17]. MVHP is mainly characterized by BRAF V600E mutation (substitution of valine for glutamic acid in position 600) and CIMP-H (CpG island methylator phenotype with high methylation status), while GCHP harbors KRAS mutation (often missense substitutions at glycine codons 12 or 13) in 50% of cases and CIMP-L (CpG island methylator phenotype with low methylation status) [18,19]. The consequence of BRAF or KRAS mutations is the activation of the MAP kinase signaling pathway, which inhibits apoptosis and promotes the proliferation of tumoral cells.

### 2.2 SSLs

SSL is the second most common SP subtype, representing 10–20% of SP [7,15,20]. The majority of SSLs (75–80%) is found in the proximal colon, including the cecum, ascending, or transverse colon [9,21]. Among SP, they show the highest potential for malignant transformation. From a genetic point of view, SSL is associated with the serrated neoplasia pathway [22].

It is characterized by deep crypt distortion (Figure 1b), because the proliferation zone moves to the crypt side, causing horizontal growth along the *muscolaris mucosae*, dilation of the crypt base, serrations extending into the crypt base, and asymmetrical proliferation. The presence of at least one of these features in sufficient to define a crypt as abnormal, as well as this feature must be unequivocal in ≥1 crypt [9].

In the course of a colonoscopy, SSAs are pale, usually larger than 5 mm, flat or only slightly raised and irregular borders. The majority of SSA produces a large amount of mucin and is surrounded by a rim of “debris.” However, the diagnosis cannot be based on the size, the location, and the endoscopic appearance of the lesion.

### 2.3 SSLs with dysplasia

The occurrence of dysplasia within a sessile serrated lesion is a rare condition (Figure 1c). Usually, dysplasia is more heterogeneous than in conventional adenomas, and for this reason, the distinction between low- and high-grade forms is not indicated. In general, it is considered a transient step during progression to cancer.

### 2.4 TSAs

TSAs are the rarest subtype of SP, accounting for only 1% of SP [21]. TSAs usually present as large polypoid lesions located in the distal colon and rectum [21]. Histologically, they present “sawtooth” crypts arranged in a slit-like pattern. TSA is classically composed of tall columnar cells with intensely eosinophilic cytoplasm and penicillate nuclei [9]. TSA initially present *KRAS* and *BRAF* mutations, which cause uncontrolled cellular proliferation. In a recent study, Hashimoto et al., evaluated the WNT mutational status of TSAs and described the malignant evolution from precursor polyps to TSAs [23]. In this study, *RNF43*, *APC*, or *CTNNB1* mutations were exclusively present in TSAs [23]. The occurrence of epigenetic silencing of DNA repair genes, with the subsequent accumulation of mutations, leads the transformation into CRC.

### 2.5 Unclassified serrated adenomas

This is a “basket” category including serrated dysplastic cases whose morphological features do not fall into any of the categories described earlier.

## 3 Serrated lesions and CRC

Two different molecular pathways underlie the colonic neoplastic transformation: the conventional and the serrated neoplasia pathways. They both are driven by the accumulation of specific molecular alterations, corresponding morphologic features and clinicopathological manifestations [24–26].

The conventional model, or the so-called adenoma-carcinoma sequence, has been proposed by Vogelstein et al. in 1988 [27] and is characterized by adenomas (including tubular or tubulovillous adenomas) as the only precursor lesions capable to give rise to CRC [28,29]. However, over the last ten years, it has been demonstrated that approximately 15–30% of all CRCs develop from the alternative/serrated pathway [25,30].

The association between SP and CRC has been demonstrated at several levels. First, both low- and high-grade dysplasia and adenocarcinoma have been found in the context of SSA lesions [31]. Also, peculiar *BRAF* mutations and the presence of *CpG island methylation phenotype* are characteristics of serrated lesions and are found, with an increasing frequency, in HPs, SSL without dysplasia, and SSL with dysplasia. These genetic changes are rarely present in conventional adenomas but are found in more than 15% of CRCs [32,33]. They occur in a structured and well-defined pathway, which is known as the serrated-neoplasia pathway [22]. The molecular and genetic progression along this pathway parallels the progression of SP to dysplasia and adenocarcinoma, according to a stepwise modality.

The time required for the malignant transformation of SP is unknown. Lash et al. observed that the presence and grade of dysplasia in the context of SP was age-related [31]. This led the authors to hypothesize that the malignant transformation needs a long time frame from 10 to 15 years [31]. However, it has been shown that this progression can also occur rapidly [34]. Interestingly, the majority of interval CRCs shows a genotype consistent with the serrated neoplasia pathway [35,36]. This evidence suggests that SP could be considered as responsible for interval CRCs. Whether this occurs because SPs are easily missed during a colonoscopy [37,38] or because SPs develop rapidly and present an accelerated malignant transformation, it is unknown.

## 4 Association between gut microbiota and serrated pathway

Important players of human health and disease are microbial species that colonize the gastrointestinal tract [39,40]. A different bacterial population can be identified in the human gut system, with a particular incidence of *Firmicutes* (30–50%), *Bacteroidetes* (20–40%), and *Actinobacteria* (1–10%) [40]. The microbial gut composition can significantly contribute to and affect several human diseases, as well as the carcinogenesis of the colorectum [40–42]. The presence of *Fusobacterium* species, particularly of *Fusobacterium nucleatum* (*F. nucleatum*), interacting with genetic changes and the innate immune system has been linked with the development of CRC, as demonstrated by its overabundance in colorectal tumor tissues when compared with the adjacent normal tissues [43,44].

The role of the Gram-negative *F. nucleatum* in the serrated pathway is not fully understood. Studies have proven *F. nucleatum* increased levels in MSI and CIMP molecular subsets, and in TA and SSL lesions, highlighting its putative involvement in the serrated pathway [44–47]. Ito et al. showed that *F. nucleatum* was associated with premalignant lesions characterized only by CIMP-H status and large tumor size [45]. Furthermore, they demonstrated that *F. nucleatum* gradually increases in SSLs from the sigmoid colon to the cecum [45]. Then, Park et al. showed a similar *F. nucleatum* abundance in TA and SSL but lower when compared with the CRC group [46]. Although these data demonstrate the *F. nucleatum* contribution to the serrated pathway, more studies are necessary to characterize its role in the carcinogenetic sequence.

## 5 MicroRNAs and long non-coding RNAs: other potential biomarkers of the serrated pathway

The important role of non-coding RNAs (ncRNAs), such as microRNAs (miRNAs) and long non-coding RNAs (lncRNAs), as biomarkers is increasingly growing in the diagnosis and management of CRCs [47]. miRNAs and lncRNAs regulate gene expression at translational and post-translational levels [48]. In addition, their alteration, in terms of low or high expression, has been associated with the traditional adenoma-carcinoma and serrated-carcinoma sequences [49–51].

Several ncRNAs profiling-based studies have identified specific miRNAs/lncRNAs associated with the serrated lesions, whose differentially expression has allowed, in addition to the canonical tests, to better distinguish between the different CRCs subtypes and serrated and nonserrated lesions. Examples are miR-335, -222, and -21, significantly differentially expressed in nonserrated when compared with serrated lesions [51], and miR-125b and miR-320a described as specific biomarkers predictive of the evolution to CRC through the serrated pathway [51]. The well-known miR-31, already associated with *BRAF* mutation in CRC, is involved in the progression of the serrated lesions and has been frequently found overexpressed in SSLs [49,52]. Recently, Kanth and collaborators have identified a serrated-specific miRNA signature using a small RNA sequencing approach. In particular, among all the differentially expressed detected miRNAs, miR-31-5p and -135B-5p have been described to be significantly overexpressed in SSLs when compared with HPs [49].

Regarding lncRNAs, several studies have demonstrated their role in the serrated pathway [50,53]. Chen et al. have identified dysregulated lncRNAs to be able to classify the 888 CRC samples analyzed into five distinct molecular subtypes [53]. In a recently published study, de Bony and collaborators have detected 282 lncRNAs corresponding to CRCs heterogeneity [50].

Thus, these findings suggest that miRNAs and lncRNAs play an important role in CRC and the serrated pathway. Further researches will be necessary to explore ncRNAs role in CRCs not only to distinguish the heterogeneous CRCs types but also for rapid and noninvasive diagnosis, and to reveal unknown mechanisms of the pathogenesis of serrated lesions.

## 6 Prevalence of serrated lesions

The prevalence of SP, namely the rate of detection of at least one SP among patients undergoing a colonoscopy, has been studied by several authors. The interest in this data goes beyond a pure epidemiological sake. Indeed, the adenoma detection rate (ADR) is considered nowadays as the main indicator of the quality of a colonoscopy, as it is negatively correlated with the occurrence of interval CRCs [54]. For example, an ADR ≤ 25% in colonoscopies performed on male patients older than 50 years is indicative of the low-quality of the procedures [55]. As missed SP is probably at the origin of interval CRCs, the SP detection rate naturally promotes itself as a quality indicator of colonoscopy, being potentially even more accurate than ADR.

Studies trying to address the prevalence of SP have shown variable results [56,57]. This variability mainly depends on the period of the study (e.g. before/after WHO classification), the awareness of the risk of cancer of SP, and the quality of pathologic and endoscopic examination. Indeed, SPs are at risk of being missed, in consideration of their morphology (flat or sessile) and their location (often in the right colon) [2,3]. Great variability in the proximal SP detection has been shown among the endoscopists, with low-detectors (referred to ADR) showing also the lowest detection rate of SP [58–60]. In addition, some of the variability is also due to the pathologist experience, as the interpathologist agreement on the diagnosis of SP is moderate [61].

Studies on autopsies have defined a wide range for the prevalence, from 6% to 29% [56,62]. However, the contribution from these studies to the daily practice remains limited, due to different methodologies.

Recently, Ijspert and colleagues published the results of a European multicenter study based on data from five screening cohorts [61]. The quality of this study relies on the expertise of endoscopists (the lowest ADR was around 30%) and the expertise of the pathologists, who were gastrointestinal-dedicated pathologists. The detection rate of SP varied between 15% and 27.2%, while SSAs were detected in the 2–6% of the patients. The rate of SPS varied between 0.03% and 0.5% [61].

Interestingly, the low prevalence of SP within cohorts undergoing a screening colonoscopy after a positive occult fecal blood test [61,63] support data from a recent study showing that this screening test has no role in the detection of SP, which rarely bleed [64].

## 7 Risk factors for SP

Some factors have been studied as potential influencers of the development of SP (Table 1). The diagnosis of SP is age-correlated, and the median age of presentation of SP is 60 years [21]. With regard to gender, SPs are equally found in females as males [21]. However, some studies indicate a higher prevalence in females [56].

**Table 1:** Factors associated with the development of serrated lesions

Factor	Strength of association	Risk
Smoking (≥30 packs/year)	OR, 2.52 (95% CI, 2.29–2.78)^a^	Increased
Alcohol intake (>14 g/day in males; 7 g/day in females)	OR, 1.33 (95% CI, 1.24–1.43)^a^	Increased
Obesity (body mass index >35)	OR, 1.34 (95% CI, 1.23–1.46)^a^	Increased
Vitamin D – intake (4th quartile)	OR, 0.92 (95% CI, 0.86–0.98)^a^	Reduced
Marine omega-3 fatty – intake (4th quartile)	OR, 0.90 (95% CI, 0.84–0.96)^a^	Reduced
Regular acetylsalicylic acid use	OR, 0.72 (95% CI, 0.59–0.87)^a^	Reduced

OR, odds ratio.

^a^Data from He et al. [68]

Similar to other colorectal polyps, SPs are more frequently found in the Western world, probably because endoscopy is more accessible [56].

The association between several lifestyle habits and SP factors has been investigated. Smoking is strongly associated with the risk of developing SP, especially SSAs and HPs [65,66]. In particular, Anderson et al. observed an increased risk of SSA in heavy smokers (more than 20 packs/year) [67]. Interestingly, the association seems to be stronger with SP than conventional adenomas [68].

Alcohol intake is related to a high risk of developing SP especially, in the left colon and rectum, although evidence is limited to heavy drinkers (>14 g/d in male; 7 g/d in female) [69]. Some authors even investigated the risk linked to different beverages, without reaching any significant conclusion [70]. Obesity (BMI > 30) has been described as a risk factor for SP in some studies, although others did not find any correlation [56,67,71].

Dietary factors such as red meat and fatty acid consumption seem to increase the risk of SP [67,71]. Physical activity and the intake of folate, calcium, fruit, and vegetables do not affect the occurrence of SP, while a high intake of vitamin D and marine omega-3 fatty acid seems to have a protective role [65,68].

Among medications, aspirin and other non-steroidal anti inflammatory drugs have been investigated and could have a protective role. Bouwens et al. demonstrated a decreased risk with the use of aspirin, especially for right-sided lesions [69].

Based on this evidence, Bouwens et al. proposed a score to predict the risk of detection SP in patients undergoing colonoscopy. This score takes into account four variables, including age (>50 years), a previous diagnosis of SP, the smoker status, and the assumption of aspirin [69].

## 8 Risk of CRC and surveillance

Determination of the long-term risk of developing CRC in patients diagnosed with an SP is essential to establish an effective surveillance program for these patients [72]. Lazarus and colleagues showed the occurrence of a metachronous CRC in the 5% of patients diagnosed with an SSL presenting dysplasia [73]. In this group of patients, Teriaky et al. found a similar risk at 5 years [74]. A higher risk was described by Lu et al.; they found CRC in 12.5% of patients being previously diagnosed with an SSL, independently from the presence of dysplasia [13]. On the contrary, Burnett-Hartman and colleagues did not find an increased risk of advanced CRC diagnosis in the 5 years following the removal of serrated lesions [75]. These studies, however, suffer from a limited sample size. Studies reporting long-term results after SP removal are still awaited and recommendations on the endoscopic surveillance in these patients are, at the moment, mainly based on expert opinions.

A panel of experts from the US recommends that patients diagnosed with an HP should receive a colonoscopy in 10 years unless HPs are found proximally to the sigma [10]. In this case, patients should receive a colonoscopy within 5 years if HPs are ≥4 or larger than 5 mm. Patients with SSL or TSA need a colonoscopy in 5 years if the lesions are small (<10 mm) and less than 3; otherwise, the colonoscopy should be anticipated at 3 years or even before, especially in case dysplasia is found [10].

European Guidelines consider at high-risk patients diagnosed with SP of large size (≥10 mm) or presenting dysplasia [76]. These patients should receive surveillance colonoscopy within 3 years. Patients with SPS should be addressed to genetic counseling. Other patients with SP should receive a colonoscopy in 10 years [76].

Serrated polyposis syndrome (SPS) is characterized by the contemporary presence of multiple serrated lesions. According to WHO criteria, SP is defined as:

(a) the presence of at least 5 SP proximal to the sigmoid colon, with ≥2 larger than 10 mm
(b) the presence of any number of SP proximal to the sigmoid colon in an individual who has a first degree relative with SP
(c) The presence of more than 20 SP of any size is distributed throughout the colon.

Patients with SPS have a higher risk of developing CRC [16,77] and therefore require strict surveillance [10]. First-degree relatives of these patients also present a high risk of CRC and should undergo a tailored colonoscopy surveillance program [10].

In conclusion, since the inclusion in 2010 of SP in the WHO classification, a large corpus of evidence regarding the epidemiology of these lesions has become available in the literature. In particular, the interest in tumor molecular changes and its connections to the external exposures and the tumor behavior is increasing. In this area of research, we think that molecular pathological epidemiology can lead to a better understanding of tumor natural history, by providing a better comprehension of the pathogenic processes and help tailoring personalized prevention strategy and therapy [78–79]. Further studies in these fields are needed. Another epidemiological aspect to focus is that serrated lesions of the colorectum account for around one-fifth of all precancerous lesions of the large bowel: proper identification, treatment, and surveillance of these lesions play a primary role within an adequate cancer prevention program. The current data suggest that a detection rate of serrated lesions ≥15% should be expected in screening colonoscopies. The detection rate of serrated-lesions, and in particular of SSL lesions, need to be evaluated as a quality indicator of colonoscopy. High-quality longitudinal studies are needed to identify the long-term risk of CRC in patients with SP, to define an evidence-based surveillance program, which is currently suggested on the base of expert opinions.
